# Resveratrol inhibits benzo[*a*]pyrene–DNA adduct formation in human bronchial epithelial cells

**DOI:** 10.1038/sj.bjc.6601898

**Published:** 2004-05-25

**Authors:** G Berge, S Øvrebø, I V Botnen, A Hewer, D H Phillips, A Haugen, S Mollerup

**Affiliations:** 1 Department of Toxicology, National Institute of Occupational Health, PO Box 8149 Dep, Oslo N-0033, Norway; 2 Section of Molecular Carcinogenesis, Institute of Cancer Research, Brookes Lawley Building, Cotswold Road, Surrey SM2 5NG, UK

**Keywords:** DNA adducts, cytochrome *P*450, human bronchial epithelial cells, metabolites, PAH, real-time RT–PCR, resveratrol

## Abstract

Resveratrol (*trans*-3,4′,5-trihydroxystilbene), a phytoalexin present in various plants and foods, has in several *in vitro* and *in vivo* studies demonstrated cancer chemopreventive and chemotherapeutic potential. We investigated the *in vitro* effect of resveratrol on benzo[*a*]pyrene (B[*a*]P) -induced DNA adducts in human bronchial epithelial cells. This was compared to the effect of resveratrol on the expression of the cytochrome P450 (CYP) genes CYP1A1 and CYP1B1 and the formation of B[*a*]P metabolites. Exposure of BEAS-2B and BEP2D cells to B[*a*]P and increasing concentrations of resveratrol resulted in a dose- and time-dependent inhibition of DNA adduct formation quantified by ^32^P-postlabelling. Supporting this result, resveratrol was shown to inhibit CYP1A1 and CYP1B1 gene expression, as measured by real-time reverse transcriptase—polymerase chain reaction. Also, a significant correlation was found between the number of DNA adducts and the mRNA levels of these genes. Using HPLC analysis, a concomitant decrease in the formation of B[*a*]P-derived metabolic products was detected. In conclusion, these data lend support to a chemopreventive role of resveratrol in polycyclic aromatic hydrocarbon-induced carcinogenesis.

Lung cancer is among the most frequent causes of cancer death in the western world today and the global incidence is increasing (Bilello *et al*, 2002; Kuper *et al*, 2002). Lung cancer is therefore expected to continue to have a major impact on human health throughout the next decades. The role of tobacco smoke as a major aetiologic factor in this malignancy is well established. Urban air pollution and occupational exposure may also increase the risk of lung cancer (Bilello *et al*, 2002). Approximately 25–30% of adults in western populations are active smokers, and the number is increasing in developing countries (Peto *et al*, 1996). The fact that in the US and in some European countries, more than 40% of new lung cancer cases occur in former smokers, merits increased focus on studies to prevent the development of the disease (Tong *et al*, 1996).

Polycyclic aromatic hydrocarbons (PAH) are potent tobacco-smoke carcinogens and occur in complex mixtures in the environment (Hecht, 2003). Benzo[*a*]pyrene (B[*a*]P) is a major constituent of these mixtures and has been widely used as a model compound in studies of PAH-induced carcinogenesis. B[*a*]P is metabolically activated by enzymes in the cytochrome P450 (CYP) system to reactive electrophilic diolepoxides that are capable of binding to DNA (Denissenko *et al*, 1996; Hecht, 2003). CYP1A1 and CYP1B1, which are major PAH bioactivating enzymes, are induced by PAH in human lung tissue (Ding and Kaminsky, 2003). Thus, inhibition of PAH bioactivation and subsequent reduction of DNA adduct formation may be an important step in the prevention of smoking associated lung carcinogenesis.

Although the confounding effect of alcohol drinking on lung cancer development is controversial and may be dose-dependent, epidemiological studies have indicated that wine consumers may have a lower risk of developing this cancer compared with consumers of other beverages (Prescott *et al*, 1999; De Stefani *et al*, 2002). Resveratrol (*trans*-3,4′,5-trihydroxystilbene), a diphenolic phytoalexin, is present in high concentrations in red wines, as well as in berries and nuts (Fremont, 2000). In addition to a possible beneficial effect of resveratrol on the risk of coronary heart disease (Renaud and de Lorgeril, 1992; Hung *et al*, 2000), resveratrol has been suggested to have cancer chemopreventive potential by interfering with many cellular pathways. The compound has been shown to block tumorigenesis in a mouse model and to inhibit tumour growth and neovascularisation in rodents (Jang *et al*, 1997; Carbo *et al*, 1999; Kimura and Okuda, 2001). Besides the antiproliferative effects, induction of apoptosis has been demonstrated both *in vitro* and *in vivo* (Carbo *et al*, 1999; Mgbonyebi *et al*, 1998; Ahmad *et al*, 2001; Narayanan *et al*, 2003). Resveratrol has also been shown to modulate the expression of genes involved in the bioactivation of PAH (Ciolino and Yeh 1999; Mollerup *et al*, 2001).

In the present study, the effect of resveratrol on B[*a*]P-derived DNA adducts was investigated in the immortalised human bronchial epithelial cell lines BEAS-2B and BEP2D. Quantitative analysis of DNA adducts was compared to the effect of resveratrol on the expression of CYP1A1 and CYP1B1 and on the formation of B[*a*]P-derived metabolites. A time- and dose-dependent inhibition of B[*a*]P-induced DNA adduct formation by resveratrol was found. This correlated with the levels of CYP1A1 and CYP1B1 expression and concentration of B[*a*]P metabolites. A long-term effect of resveratrol on B[*a*]P-metabolite formation was found.

## MATERIAL AND METHODS

### Chemicals

Unless otherwise stated all chemicals were from Sigma (St Louis, MO, USA).

### Cell lines and culture conditions

The immortalised human bronchial epithelial cell lines BEP2D and BEAS-2B were kindly provided by Drs JC Willey and CC Harris, respectively. The cells were maintained in LHC-9 medium (BioFluids, Rockville, MD, USA) containing 0.45 *μ*g ml^−1^ bovine serum albumin, at 37°C in a humidified 5% CO_2_ atmosphere. In the experiments, 80–90% confluent cell cultures were treated with 0–50 *μ*M resveratrol (Sigma, St Louis, MO, USA) in the presence of 1 *μ*M B[*a*]P (Sigma) for 24–120 h. After incubation, the cell culture medium was collected and kept at −20°C until further analysis (high-performance liquid chromatography, HPLC). The plates were washed with cold phosphate-buffered saline, treated with trypsin (Sigma) and the cell pellet stored at −70°C for RNA isolation and DNA adduct measurements. Solutions of resveratrol and B[*a*]P were made in 100% DMSO. During the experiments, the final concentration of DMSO was 0.1%. Experiments were performed in replicates of three and repeated several times, with similar results.

### Aromatic/hydrophobic DNA adducts

DNA was extracted from cell pellets. Aromatic/hydrophobic DNA adducts were quantified by ^32^P-postlabelling analysis with the nuclease P_1_ modification as described previously (Phillips *et al*, 1990). Each sample was measured in parallel.

### Quantitative real-time reverse transcription–polymerase chain reaction (RT–PCR)

Total RNA was extracted from cells by the TRIzol reagent (Invitrogen). mRNA was reverse transcribed by the aid of the 1st Strand cDNA Synthesis Kit for RT-PCR (AMV) using random primers (Roche Diagnostics, Basel, Switzerland). PCR primers (DNA Technology, Science Park Aarhus, Denmark) were designed to span introns to avoid amplification from traces of possible DNA contamination in the RNA isolation. Primers were checked for specificity by Blast search CYP1A1 forward, 5′-CAT CCC CCA CAG CAC AAC A-3′; CYP1A1 reverse, 5′-CAG GGG TGA GAA ACC GTT CA-3′; CYP1B1 forward, 5′-CTG GAT TTG GAG AAC GTA CCG-3′; CYP1B1 reverse, 5′-TGA TCC AAT TCT GCC TGC AC-3′; *β*-actin forward, 5′-GCG AGA AGA TGA CCC AGA TCA-3′; *β*-actin reverse, 5′-GAT AGC ACA GCC TGG ATA GCA A-3′. These primer pairs give rise to PCR products of 152, 143, and 76 bp, respectively. During establishment of the real-time PCR method, PCR products were subjected to gel electrophoresis to ensure purity and correct fragment size. Quantitative analysis of the specific expression of various genes was performed by real-time PCR, on an ABI PRISM 5700 (Applied Biosystems, Foster City, CA, USA) with SYBRgreen I (40 cycles of 95°C 15 s, 60°C 1 min). The amount of target cDNA in each sample was established by determining a fractional PCR threshold cycle number (*C*_t_), and estimated by interpolation from a standard curve. The standard curve was made from known amounts of the corresponding product with the same primer sets, and was run on each PCR plate. The expression levels of CYP1A1 and CYP1B1 were normalised to the expression of the *β*-actin gene.

### High-performance liquid chromatography

For the analysis of B[*a*]P metabolites, culture medium (3 ml) from exposed cells was collected and stored at −20°C until further processing. The medium was diluted to 10 ml in H_2_O, applied to a preconditioned (5 ml methanol and 10 ml water) Sep-Pak C_18_ cartridge (Millipore Corporation, Milford, MA, USA), followed by a wash with H_2_O (10 ml), and eluted with 100% methanol (5 ml). The methanol eluate was evaporated to dryness at 45°C under a nitrogen stream and resolubilised in 100 *μ*l of 100% methanol. HPLC separation of B[*a*]P metabolites was performed on a Nova-Pak C_18_ 3.9 × 150 mm^2^ column (Waters, Milford, MA, USA) with a Waters 625 LC System, equipped with a LC 240 fluorescence detector (Perkin-Elmer, Beaconsfield, UK). The B[*a*]P metabolites were separated by a linear gradient of 30–100% methanol in H_2_O for 40 min. For the quantitative determination of B[*a*]P tetrols, the following fluorescence conditions were used: 0 min, ex 380/em 431; 0.5 min, ex 341/em 381; 20 min, ex 253/em 410; 27 min, ex 380/em 431. The concentration of B[*a*]P metabolite standards was determined by ultraviolet absorbance of the compounds dissolved in ethanol and using extinction coefficients from the NIH Chemical Carcinogen Repository (Midwest Research Institute, Kansas City, MO, USA).

### Statistical analysis

The effect of resveratrol on the formation of DNA adducts, CYP expression and concentration of B[*a*]P-tetrol I-1 was investigated in four separate linear regression models. As cell line affected the level of response variables, it was included as a variable in all models. To achieve uniform variance throughout the data set, the response variables were log transformed in the analyses. Exposure time was considered a class variable. The concentration of resveratrol was treated as a continuous variable. The global model included resveratrol concentration, length of exposure, and the intercept between these two factors, and a backward stepwise method with 5% alpha level to exclude insignificant parameters. All analyses were carried out in R version 1.8.0 (Anon, 2003).

## RESULTS

### Effect of resveratrol on DNA adducts

**Figure 1:**
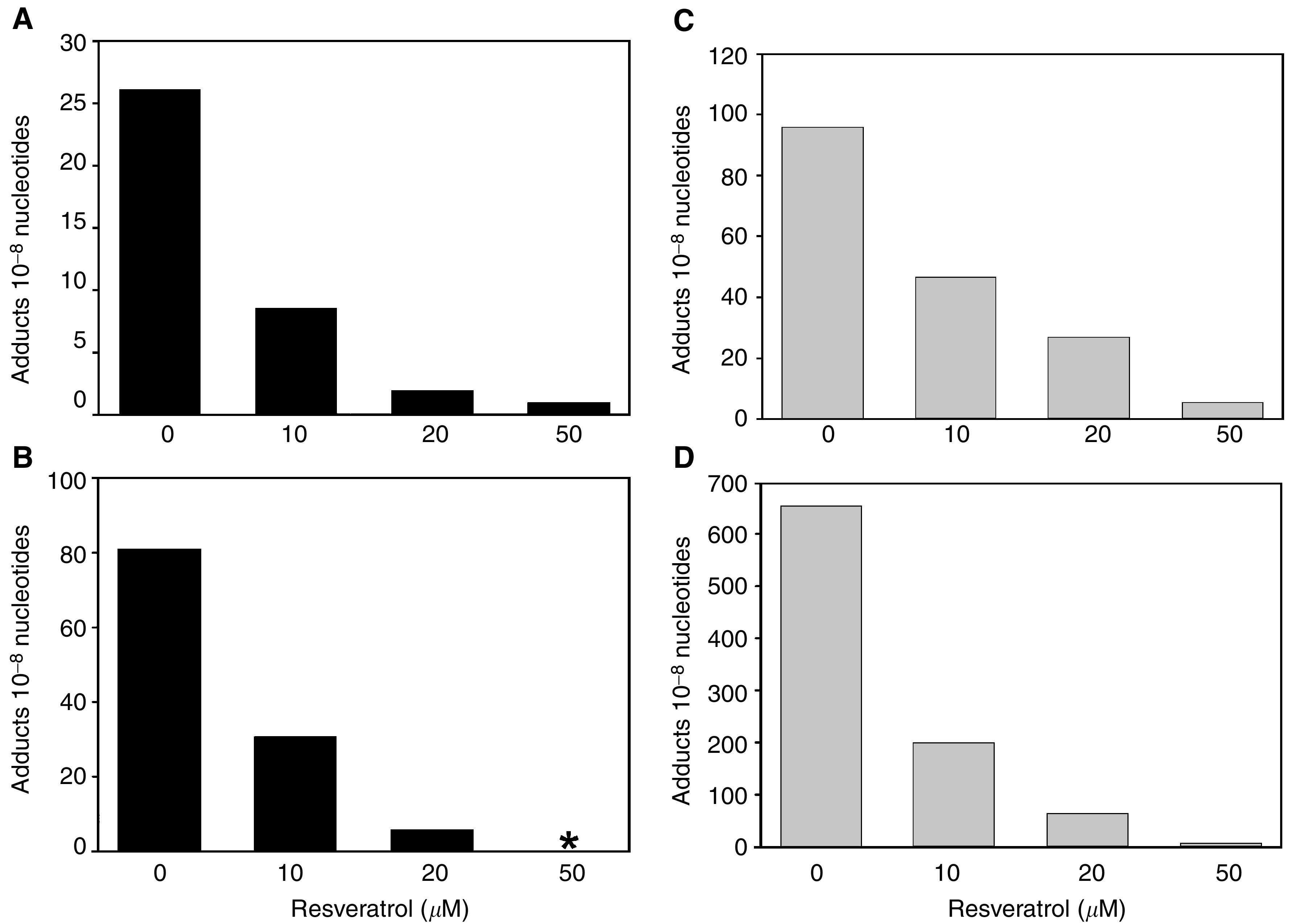
B[*a*]P–DNA adducts measured by ^32^P-postlabelling in BEAS-2B (**A** and **B**) and BEP2D cells (**C** and **D**). The cells were treated with 1 *μ*M B[*a*]P and increasing amounts of resveratrol as indicated and incubated for 24 h (**A** and **C**) and 72 hr (**B** and **D**). Columns represent the mean of two measurements from a representative assay. ^*^Not performed.

The immortalised human epithelial cell lines BEAS-2B and BEP2D were incubated with a combination of 1 *μ*M B[*a*]P and increasing amounts of resveratrol for 24 or 72 h. Treatment of both cell lines with B[*a*]P in the absence of resveratrol resulted in a significant formation of DNA adducts, and the level was higher in BEP2D than in BEAS-2B (Figure 1

). Cotreatment with 10–50 *μ*M resveratrol resulted in a dose-dependent inhibition in the number of DNA adducts (F=50.3, df=1, *P*<0.0001, for both lines combined). Incubation of BEAS-2B with 1 *μ*M B[*a*]P alone resulted in 26.1 and 80.9 adducts 10^−8^ nucleotides after 24 and 72 h, respectively. At 10 *μ*M resveratrol, these levels were reduced by 67 and 62% (Figure 1A and 1B). In BEP2D cells incubated with 1 *μ*M B[*a*]P, a level of 95.5 adducts 10^−8^ nucleotides was measured after 24 h, which increased to 652.1 after 72 h (Figure 1C and 1D). In the presence of 10 *μ*M resveratrol, a 51% decrease was found after 24 h (Figure 1C). At 10 *μ*M resveratrol, the high number of adducts formed after 72 h was reduced by 70% (Figure 1D). The inhibiting effect of resveratrol was more pronounced after 72 than 24 h (F=5.1, df=1, *P*=0.047, for both cell lines combined).

### Quantitative real-time RT–PCR

**Figure 2:**
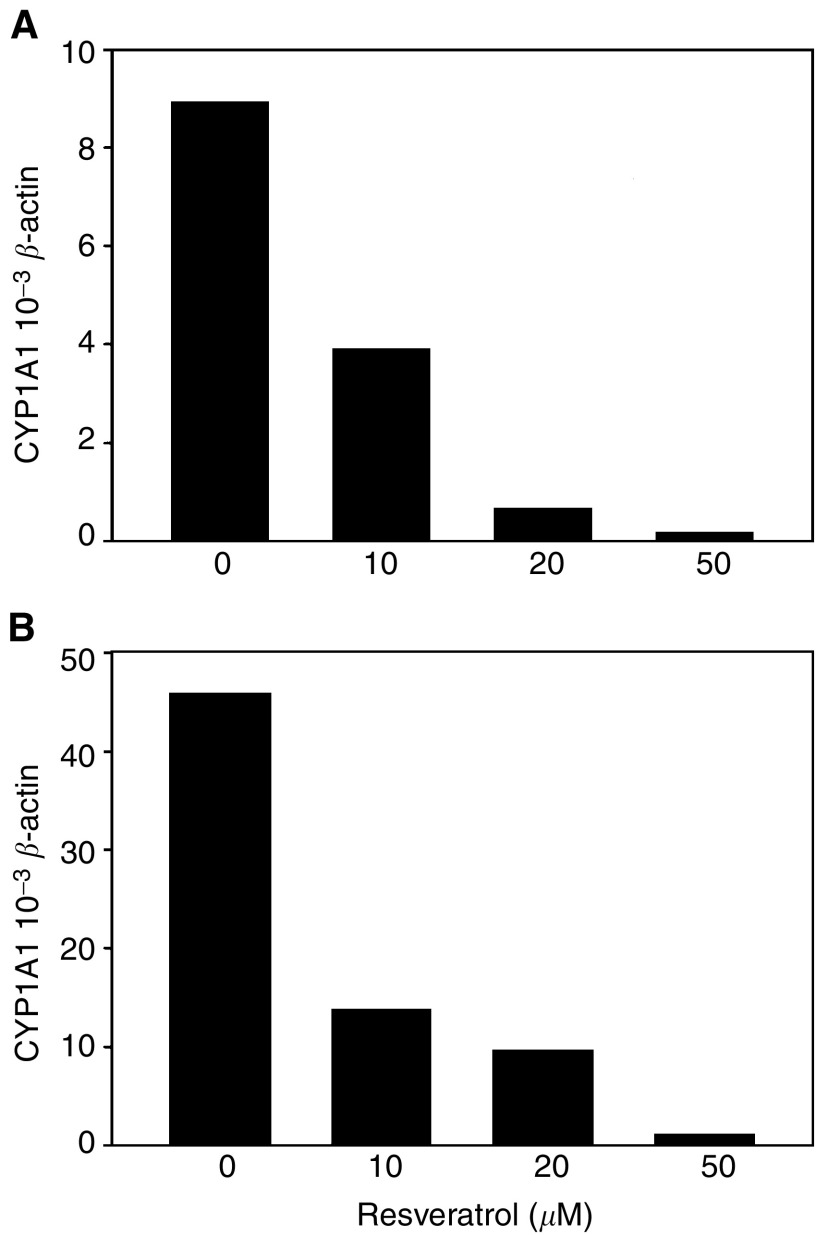
Real-time RT–PCR measurement of CYP1A1 expression relative to the expression of *β*-actin in BEAS-2B cells. Cells were treated with 1 *μ*M B[*a*]P and increasing amounts of resveratrol for 24 h (**A**) and 72 h (**B**). Columns represent the mean of repetitive PCRs from an experiment in which cells from two parallel Petri dishes were pooled during RNA isolation.

The expression levels of CYP1A1 and CYP1B1 mRNA were determined in the human bronchial epithelial cell lines by real-time RT–PCR and normalised to the expression of *β*-actin. Data for BEAS-2B are shown in Figure 2

. The BEP2D cells responded to B[*a*]P and resveratrol exposure similarly as reported in Mollerup *et al* (2001). CYP1A1 mRNA increased from 24 to 72 h in both cell lines exposed to B[*a*]P (F=35.3, df=1, *P*=0.0001). Cotreatment with increasing concentrations of resveratrol resulted in a decreased level of CYP1A1 mRNA (F=39.3, df=1, *P*<0.0001, for both cell lines combined). In BEAS-2B cells, 10 *μ*M resveratrol reduced CYP1A1 by 56 and 70% after 24 and 72 h, respectively. For 50 *μ*M resveratrol, the reduction was 97% at both time points (Figure 2A and 2B). In BEAS-2B cells, CYP1B1 mRNA appeared to be maximally induced by B[*a*]P after 24 h (data not shown). Increasing concentrations of resveratrol also inhibited CYP1B1 gene expression dose dependently at both time points (both cell lines combined: F=7.7, df=1, *P*=0.0001).

The expression levels of CYP1A1 and CYP1B1 were significantly related to the number of DNA adducts (F=19.7, df=1, *P*=0.001, and F=8.2, df=1, *P*=0.01, for CYP1A1 and CYP1B1, respectively). However, both genes are transcriptionally activated by the aryl hydrocarbon receptor (AHR), and the mRNA levels of the two genes were found to be strongly correlated.

### Effect of resveratrol on the formation of B[*a*]P metabolites

**Figure 3:**
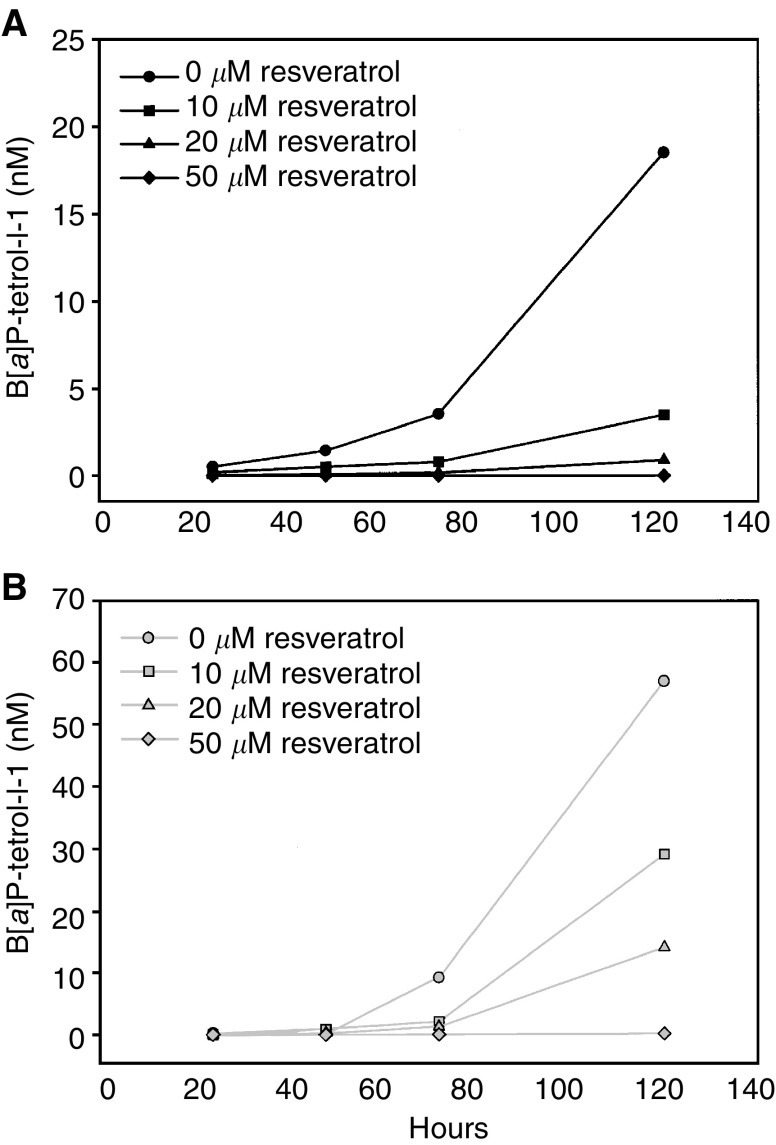
Effect of resveratrol on B[*a*]P-tetrol I-1 formation over time. The cell lines BEAS-2B (**A**) and BEP2D (**B**) were treated with a combination of 1 *μ*M B[*a*]P and 0–50 *μ*M resveratrol as indicated. The cell culture medium was collected at 24, 48, 72 and 120 h of incubation. The levels of the B[*a*]P-tetrol I-1 metabolite in the medium were measured by fluorescence HPLC. Data points represent measurement of the medium from two parallel Petri dishes pooled at the end of incubation.

B[*a*]P is metabolised into the carcinogenic diolepoxides, BPDE-I and BPDE-II. The effect of resveratrol on the formation of B[*a*]P-tetrols was measured by fluorescence HPLC in the cell culture medium. The cells were treated with 1 *μ*M B[*a*]P and 0–50 *μ*M resveratrol for 24, 48, 72 or 120 h. The hydrolysis product of the ultimate carcinogen BPDE-I, B[*a*]P-tetrol I-1, was found as the most abundant form. In the absence of resveratrol, the formation of B[*a*]P-tetrol I-1 increased steadily during the 120-h period (Figure 3

). With the addition of resveratrol, the formation of the tetrol was inhibited in a dose-dependent manner throughout the incubation period in both cell lines (F=210.1, df=1.58, *P*=0.0001). A time effect was found, where the effect of resveratrol increased with incubation time (F=4.4, df=3.58, *P*=0.003). For BEAS-2B, incubation in the presence of 1 *μ*M B[*a*]P led to an increase from 0.5 nM B[*a*]P-tetrol I-1 (24 h) to 18.5 nM B[*a*]P-tetrol I-1 (120 h). Cotreatment with 10 *μ*M resveratrol reduced the level by 60% after 24 h and 71% after 120 h. At 50 *μ*M resveratrol, the concentration of the tetrol was below detection limits at all time points (Figure 3A). The BEP2D cells showed a similar pattern of induction and inhibition, while the concentration of B[*a*]P-tetrol I-1 reached higher levels than in the BEAS-2B. In BEP2D, a concentration of 57.0 nM of the tetrol was measured after 120 h without resveratrol. At 50 *μ*M resveratrol, virtually no B[*a*]P-tetrol I-1 was detectable (Figure 3B).

The other tetrols (B[*a*]P-tetrol I-2, B[*a*]P-tetrol II-1, and B[*a*]P-tetrol II-2) showed similar trends or were below detection limits.

## DISCUSSION

The interest in chemicals effective in the reduction or inhibition of cancer is increasing and chemoprevention via nontoxic agents would greatly benefit public health. Plant-derived foods contain thousands of chemically dissimilar phytochemicals, many of which have been studied *in vitro* and *in vivo* to determine their effects on cancer risk and their mechanism of action. DNA adducts may be considered a risk factor for lung cancer, and their measurement can be used as a biomarker for PAH exposure (Phillips *et al*, 1988; Mollerup *et al*, 1999; Wiencke *et al*, 1999). In this study, we investigated the effect of resveratrol on premutagenic B[*a*]P–DNA adducts in the human bronchial epithelial cell lines BEAS-2B and BEP2D. We used ^32^P-postlabelling to quantify B[*a*]P–DNA adducts. Exposure to 1 *μ*M of B[*a*]P led to a significant induction of adducts, which was inhibited by cotreatment with 10–50 *μ*M resveratrol in a time- and dose-dependent manner. The present report shows that resveratrol can inhibit the formation of a genotoxic end product of a tobacco-smoke carcinogen in human bronchial epithelial cells. The level of DNA adducts differed between the cell lines, being higher in BEP2D than in BEAS-2B. A concentration of 10 *μ*M resveratrol showed strong inhibitory effects on the level of DNA adducts and halved the number of adducts in both BEAS-2B and BEP2D after treatment for 24 h. At 50 *μ*M resveratrol, an almost complete inhibition of DNA adduct formation in both cell lines for up to 72 h was observed. This indicates that resveratrol is a competent inhibitor of B[*a*]P adduct formation irrespective of the bioactivating capacity of the cell line. In support of our data, Revel *et al* (2003) found by a semiquantitative immunohistochemical method that resveratrol inhibited lung B[*a*]P–DNA adduct formation in mice.

Resveratrol was found to exhibit a strong dose-dependent inhibition of both CYP1A1 and CYP1B1 expression. This is in accordance with previous data, where resveratrol inhibited the expression levels of CYP1A1 and CYP1B1 in BEP2D cells exposed to B[*a*]P for 24 h (Mollerup *et al*, 2001). In this study, we show that resveratrol had a long-lasting transcriptional inhibitory effect on both genes, which correlated with a reduced capacity to metabolise B[*a*]P. We have previously shown a significant relationship between CYP1A1 expression and the level of DNA adducts in normal human lung tissue (Mollerup *et al*, 1999). Similarly, in this *in vitro* assay the inhibition of CYP1A1 and CYP1B1 expression by resveratrol could significantly explain the reduction in the number of DNA adducts. Decreased levels of CYP1A1 mRNA and protein activity and modulation of CYP1B1 expression by resveratrol has also been shown in other cell types (Ciolino and Yeh, 1999; Chang *et al*, 2000; Lee and Safe, 2001). Various molecular interactions have been proposed to be responsible for these inhibitory effects of resveratrol. Casper *et al* (1999) reported that resveratrol inhibited CYP1A1 expression by functioning as an aryl hydrocarbon receptor (AHR) antagonist. This is in contrast to studies by Ciolino and Yeh (1999) showing that resveratrol does not replace TCDD from AHR, but prevents the transformation of AHR to an activated nuclear DNA binding form. An AHR-independent post-transcriptional pathway has also been suggested, in which resveratrol increases the rate of CYP1A1 mRNA degradation (Lee and Safe, 2001). In addition, a direct inhibition of CYP1A1 enzymatic activity in cell free extracts has been shown (Chun *et al*, 1999; Ciolino and Yeh, 1999).

After exposure to B[*a*]P, the most abundant metabolite identified in the cell culture medium was B[*a*]P-tetrol I-1, a hydrolysis product of the ultimate carcinogen BPDE-I. The formation of the tetrol was inhibited dose dependently by resveratrol from 24 to 120 h. However, in the absence of resveratrol the rate B[*a*]P-tetrol I-1 formation increased throughout the same incubation period. These results indicate a prolonged inhibitory effect of resveratrol on PAH metabolism and the inhibiting effect was found to be more pronounced over time.

The total beneficial effect of resveratrol may be a result of effects on various cell systems, and the compound may inhibit all of the initiation, promotion, and progression phases of carcinogenesis. The ring structure of resveratrol makes it an antioxidant (Ray *et al*, 1999). In addition to CYP1A1 and CYP1B1, resveratrol may affect mRNA levels of genes involved in phase II drug-metabolising enzymes (Mollerup *et al*, 2001). Resveratrol has been shown to disturb cell cycle regulation and induce apoptosis. Both p53-dependent and -independent induction of apoptosis by resveratrol has been shown in various cell lines (Huang *et al*, 1999; Ahmad *et al*, 2001; Mahyar-Roemer *et al*, 2001; Narayanan *et al*, 2003). Apoptosis was also shown to be initiated in a tumour cell inoculation assay in rats (Carbo *et al*, 1999). Resveratrol also inhibited cell growth and modulated cell cycle in several human cancer cells (Mgbonyebi *et al*, 1998; Ferry-Dumazet *et al*, 2002). An antipromotion activity of the compound by inhibiting cyclo-oxygenase, as well as DNA polymerase and ribonucleotide reductase has been reported (Subbaramaiah *et al*, 1998; Fontecave *et al*, 1998; Sun *et al*, 1998).

Modulation of PAH procarcinogen activation either by preventing the induction of metabolising enzymes or by inhibiting enzyme activity may be an important step in the chemoprevention of cancer and, in particular, lung cancer. This work shows that resveratrol can inhibit the formation of DNA adducts in human bronchial epithelial cells *in vitro* and supports a role for resveratrol as a chemopreventive dietary constituent. Further studies are needed to clarify whether this effect can be reproduced in an *in vivo* model of lung carcinogenesis.
